# Achieving Rapid Blood Pressure Control With Digital Therapeutics: Retrospective Cohort and Machine Learning Study

**DOI:** 10.2196/13030

**Published:** 2019-03-12

**Authors:** Nicole L Guthrie, Mark A Berman, Katherine L Edwards, Kevin J Appelbaum, Sourav Dey, Jason Carpenter, David M Eisenberg, David L Katz

**Affiliations:** 1 Better Therapeutics San Francsico, CA United States; 2 Manifold, Inc Oakland, CA United States; 3 Department of Nutrition Harvard TH Chan School of Public Health Harvard University Boston, MA United States; 4 Yale-Griffin Prevention Research Center Griffin Hospital Yale School of Public Health Derby, CT United States

**Keywords:** hypertension, mobile health, mHealth, lifestyle medicine, digital therapeutics, digital medicine, machine learning, behavioral therapy

## Abstract

**Background:**

Behavioral therapies, such as electronic counseling and self-monitoring dispensed through mobile apps, have been shown to improve blood pressure, but the results vary and long-term engagement is a challenge. Machine learning is a rapidly advancing discipline that can be used to generate predictive and responsive models for the management and treatment of chronic conditions and shows potential for meaningfully improving outcomes.

**Objective:**

The objectives of this retrospective analysis were to examine the effect of a novel digital therapeutic on blood pressure in adults with hypertension and to explore the ability of machine learning to predict participant completion of the intervention.

**Methods:**

Participants with hypertension, who engaged with the digital intervention for at least 2 weeks and had paired blood pressure values, were identified from the intervention database. Participants were required to be ≥18 years old, reside in the United States, and own a smartphone. The digital intervention offers personalized behavior therapy, including goal setting, skill building, and self-monitoring. Participants reported blood pressure values at will, and changes were calculated using averages of baseline and final values for each participant. Machine learning was used to generate a model of participants who would complete the intervention. Random forest models were trained at days 1, 3, and 7 of the intervention, and the generalizability of the models was assessed using leave-one-out cross-validation.

**Results:**

The primary cohort comprised 172 participants with hypertension, having paired blood pressure values, who were engaged with the intervention. Of the total, 86.1% participants were women, the mean age was 55.0 years (95% CI 53.7-56.2), baseline systolic blood pressure was 138.9 mmHg (95% CI 136.6-141.3), and diastolic was 86.2 mmHg (95% CI 84.8-87.7). Mean change was –11.5 mmHg for systolic blood pressure and –5.9 mmHg for diastolic blood pressure over a mean of 62.6 days (*P*<.001). Among participants with stage 2 hypertension, mean change was –17.6 mmHg for systolic blood pressure and –8.8 mmHg for diastolic blood pressure. Changes in blood pressure remained significant in a mixed-effects model accounting for the baseline systolic blood pressure, age, gender, and body mass index (*P*<.001). A total of 43% of the participants tracking their blood pressure at 12 weeks achieved the 2017 American College of Cardiology/American Heart Association definition of blood pressure control. The 7-day predictive model for intervention completion was trained on 427 participants, and the area under the receiver operating characteristic curve was .78.

**Conclusions:**

Reductions in blood pressure were observed in adults with hypertension who used the digital therapeutic. The degree of blood pressure reduction was clinically meaningful and achieved rapidly by a majority of the studied participants. Greater improvement was observed in participants with more severe hypertension at baseline. A successful proof of concept for using machine learning to predict intervention completion was presented.

## Introduction

High blood pressure (BP), or hypertension, is the leading contributor of preventable death worldwide and based on the 2017 American College of Cardiology (ACC)/American Heart Association (AHA) guideline, it is prevalent among 45.6% of US adults [1]. This extraordinary prevalence is attributed, in part, to the omnipresent detrimental diet and lifestyle behaviors associated with hypertension [2,3].

The consequences of high BP have been appreciated since the 1930s, and an array of effective antihypertensive medications have been available for decades [4]. However, only half of those with hypertension have optimally controlled BP, and 16% have poorly controlled hypertension despite taking three or more antihypertensive medications [5,6].

In addition to pharmacotherapy, clinical guidelines in the United States and worldwide call for the initiation of behavioral therapy focused on lifestyle for all patients with hypertension, because it is known that lifestyle changes can directly lower BP while simultaneously improving other cardiovascular risk factors without the side effects of pharmacotherapy [1,7]. However, there is also widespread appreciation that the current health care system is unable to deliver behavioral therapies that predictably lead to sustained lifestyle changes among the massive volume of patients who need it [8,9].

As a part of a global call to address a worsening pandemic, technology companies have been asked to contribute innovative solutions that enhance BP control and reduce the burden of care on primary care systems [10,11]. In particular, digital interventions designed to treat chronic diseases, known as digital therapeutics, can be paired with remote monitoring devices to create novel means of delivering effective and highly accessible care. These same interventions can simultaneously monitor outcomes, as recent evidence demonstrated the validity and utility of BP monitoring at home [12,13].

There are numerous commercially available apps designed to aid BP management, especially BP-tracking apps, but very few of them are multicomponent behavioral interventions designed to treat hypertension and have been clinically evaluated [14-17]. The widespread availability of mobile health apps, and the difficulty patients and clinicians have in distinguishing between them, warrants more rigorous study and vetting [18].

The use of machine learning, a branch of artificial intelligence that aims to make sense of patterns within large datasets, offers the potential to further increase the effectiveness of digital interventions. For example, it can be used to predict the likelihood of a specific clinical outcome based on an individual’s unique pattern of use of the multiple components that make up a digital intervention during the course of treatment. This predictive ability holds great promise in developing interventions that are precisely targeted to the individual for optimal effectiveness. It has been argued that improving our ability to target treatment to individual patients begins by identifying and addressing unique subgroups through advanced analytic techniques like machine learning and may be the best path forward to enact precision medicine [19,20].

Mobile apps are well situated to use machine learning, because they are continuously collecting engagement and biometric data and can be programmed to change the delivery of treatment in response to outputs of machine learning algorithms. Although the application of machine learning to mobile apps holds great promise, it has not been widely applied to mobile apps targeting the root causes of hypertension.

The digital intervention assessed in this paper was developed by Better Therapeutics LLC (San Francisco, CA), a developer of prescription digital therapeutics for the treatment of cardiometabolic diseases. The goal of this article is to provide a retrospective analysis of the effectiveness of digital therapeutics in delivering behavioral therapy to patients with hypertension, resulting in a reduction of BP. In addition, a proof of concept for the use of machine learning to predict intervention completion in a manner that allows for personalized, real-time treatment plan adjustments is presented.

## Methods

### Digital Intervention

The digital intervention integrates a mobile medical app that delivers behavioral therapy with the support of a remote multidisciplinary care team. The mobile app delivers a personalized behavior change intervention including tools for goal setting, skill building, self-monitoring, biometric tracking, and behavioral feedback. The intervention is designed to support the participant’s daily efforts to reduce BP and improve overall cardiometabolic function by facilitating behavioral changes, such as planning and self-monitoring, that increase physical activity and change dietary pattern to one that is predominately made up of whole grains, fruits, vegetables, beans, legumes, nuts, and seeds. These targeted changes are consistent with well-established clinical guidelines [1,7]. Further, the app uses artificial intelligence to provide feedback and support during the intervention to enhance adherence to behavioral therapy and increase participants’ self-efficacy to make and sustain behavioral changes. Use of the app is coupled with scheduled person-to-person health coaching by phone over a 12-week treatment period. Program completion was defined as ongoing use of the intervention in week 10 or later.

The app was designed for daily use, with a typical interaction beginning in a conversational interface that prompts a participant to report their progress toward individualized behavioral goals, such as the number of plant-based meals and minutes of physical activity completed that day as well as any biometrics, such as BP or weight, that were recorded. The participant receives feedback based on the data collected and is then prompted to engage in one or more behavioral exercises. For example, they may be prompted to respond to a question from their coach to self-reflect on opportunities and barriers to meeting their weekly goals or begin a skill-learning exercise that challenges the participant to try a new method for preparing vegetables or a different strategy for incorporating exercise into their day.

Intervention participants were recruited directly through Facebook and employer-sponsored advertisements. The intervention was advertised as a 12-week program for adults who wanted to improve hypertension, type 2 diabetes, or hyperlipidemia. All enrollees who self-identified as hypertensive were provided the option to receive a free Omron 7 Series Upper Arm Blood Pressure Monitor (Omron Healthcare, Inc, Kyoto, Japan) for use throughout the intervention and to keep after the study ended. The intervention was available to individuals at no cost.

### Intervention Participants

**Figure 1:**
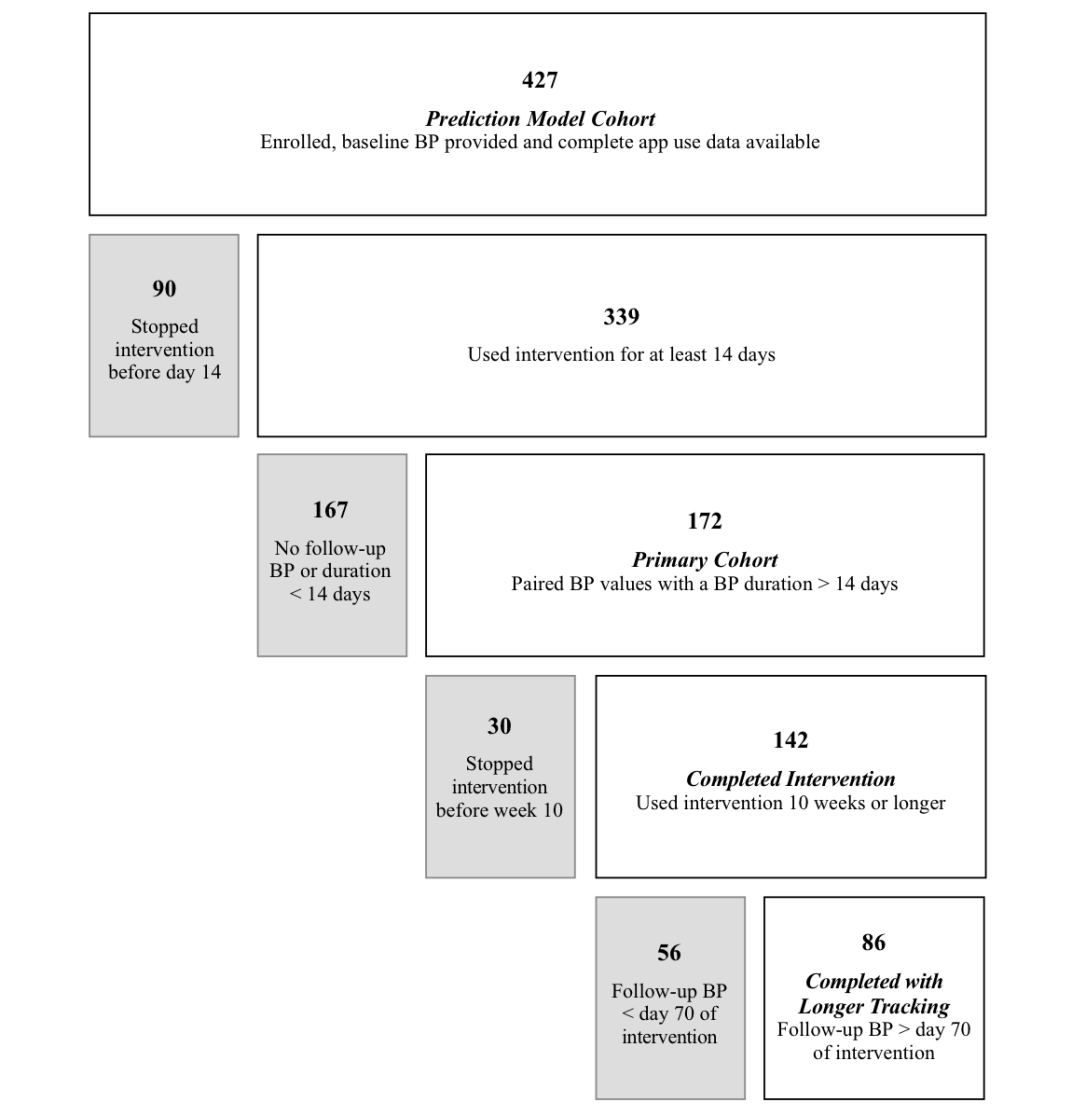
Participant flow chart. BP: blood pressure.

The intervention database was searched to identify participants with a starting BP value in the hypertensive range (≥130/80 mmHg), as defined by the 2017 ACC/AHA guideline [1], as well as participants with elevated BP who reported using antihypertensive medication. From this group of participants, analysis cohorts were identified based on engagement with the intervention (Figure 1). The intervention days were counted from day 0 (account created), with day 1 being the first full day of access to the digital intervention.

The *Primary* cohort included participants with hypertension, who provided follow-up BP values at least 14 days after the baseline. The *Completed Intervention* cohort included those who showed activity in the app in or after week 10 of the intervention. Participants were further categorized into the *Completed with Longer Tracking* cohort if, in addition to completing the intervention, their follow-up BP value was reported on or after day 70 of the intervention. The criteria for the analysis cohorts were defined prior to completing the analysis in order to explore the relationship between app engagement (*Completed Intervention*) and self-tracking (*Completed with Longer Tracking*) at the primary end point of changes in BP.

Data were identified by a unique numeric identification assigned by the system at registration; exported data included no personal identifiers. This retrospective analysis was approved and overseen by the Quorum Review Institutional Review Board [21], and a waiver of informed consent was granted for this retrospective analysis.

### Measures

#### Demographics

Participants eligible for enrollment were adults, aged 18 years or older, were living in the United States, and had a smartphone with Android or Apple operating system to access the intervention app. Within the app, participants reported their age, gender, height, weight, medical history, state of residence, and current prescription medications.

#### Blood Pressure

Participants recorded BP readings in the app at will. Each reported measurement included a value for systolic BP (SBP), diastolic BP (DBP), and date and time of measurement. Baseline and follow-up values were calculated by taking an average of all available values in 7-day intervals. Day 1 of the intervention was set as the anchor day for the baseline interval, and all values reported within the following 6 days were included in the mean. The follow-up anchor date was set as the date of the last BP value reported, and all values reported in the 6 days prior to the anchor were included in the mean. The number of days between the baseline and follow-up BP was considered the duration of change. The mean change was calculated by subtracting the mean baseline value from the mean follow-up value. BP categorization was based on published guidelines (defining elevated as 120-129/<80 mmHg, stage I hypertension as 130-139/80-89 mmHg, and stage II hypertension as ≥140/≥90 mmHg) [1].

#### Weight

In addition to reporting weight at the time of enrollment, participants had the option to track their weight using their own home scale and record it in the app at will. Body mass index was calculated by dividing the weight in kilograms by the height in meters squared.

#### Predictive Modeling

We used machine learning to generate a model to predict whether someone would complete the intervention, applying the same criteria as defined above for the *Completed Intervention* cohort. A random forest model was trained on 427 participants with complete app use data available. The random forest model was selected because it reduces overfitting of a model by taking the average of many decision trees, which is important in small data sets [22]. A supervised classification algorithm was used, since the response variable *Completed Intervention*, is binary.

**Figure 2:**
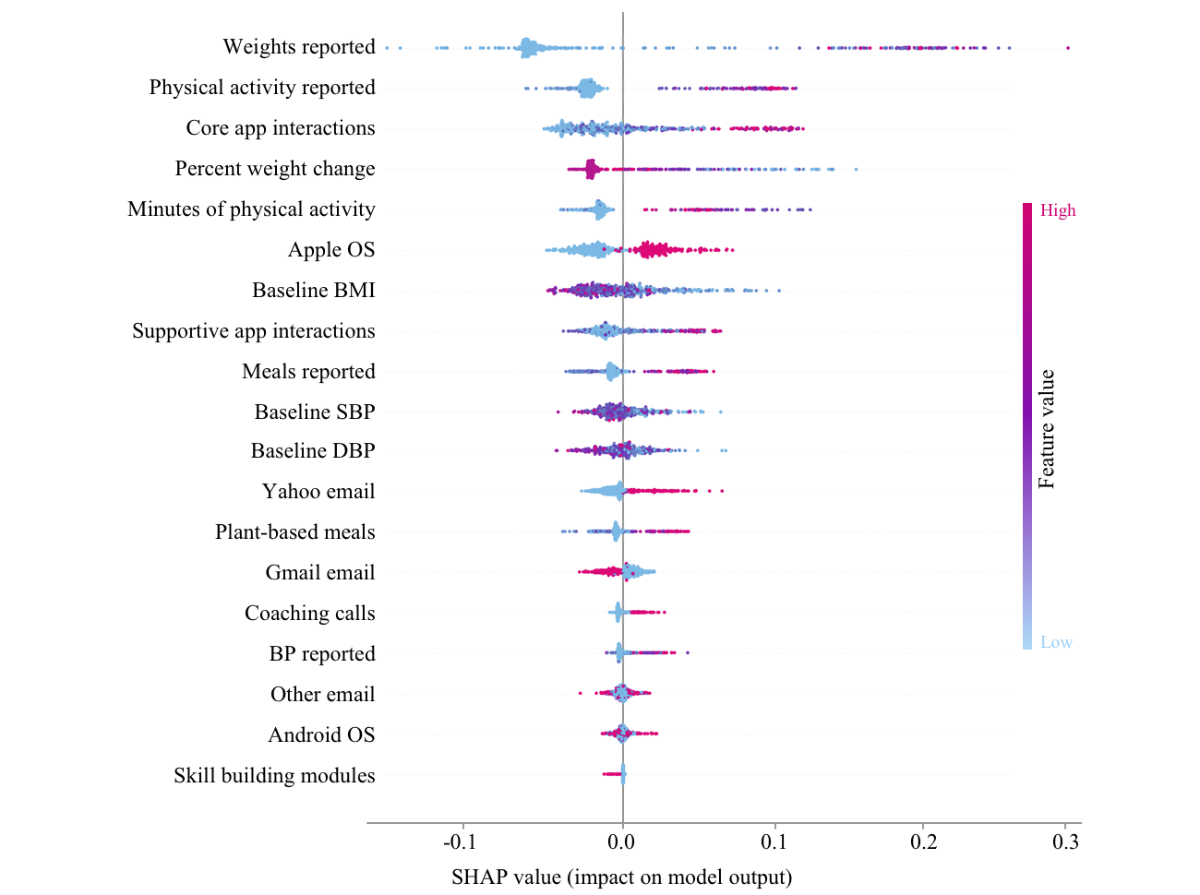
Shapley values illustrate which factors contribute most to an increased likelihood of completion (or noncompletion). Each dot represents the value of one individual participant for the feature component listed on the Y-axis. The value is represented by color (high vs low value) and by placement on the X-axis (amount of positive vs negative contribution to intervention completion). The feature list on the left is in order of contribution to the model (most to least). OS: operating system; SBP: systolic blood pressure; DBP: diastolic blood pressure; BP: blood pressure; BMI: body mass index; SHAP: Shapley Additive Explanation.

The random forest model was trained with 250 trees and a minimum of 3 samples per leaf node, as determined by hyperparameter optimization. The model included 19 features that can be grouped as follows: (1) Engagement: These features were actions related to use of the intervention, such as the count of plant-based meals logged, skill-building modules completed, or health coaching calls completed. (2) Sociomarkers: These were indicators of social conditions that an individual is exposed to or surrounded by, which can be correlated with the presence or severity of a health state, such as zone improvement plan code or availability of health care [23]. Our model incorporated the novel sociomarkers’ operating system (Android or Apple operating system) and email domain (Gmail, Yahoo, Hotmail, or Other), because we hypothesized that these sociomarkers may have predictive power. (3) Biometrics: These included the count of BP values reported, the baseline BP value, the count of weight values reported, and the percentage of weight loss. A list of all features included in the model is presented in Figure 2.

We trained random forest models on days 1, 3, and 7 of the intervention, with day 1 being the first full day of intervention engagement after the participant signs up. Development of training models at differing time points from the start day allowed us to explore the duration of engagement needed before predictive capacity emerged. For each model, only the data collected up to that day were used as features in the model. For example, in the day 3 model, we only used the engagement information collected in the first 3 days, and not beyond. For each model, the final response variable was the same—whether the patient completed the intervention. The training of the model includes a series of decision trees that evaluates data from the engagement features, sociomarkers, and biometrics, in relation to the response variable of interest—intervention completion.

We assessed generalization performance of the model by using leave-one-out cross-validation, which is a common technique for assessing model performance in samples of this size [24-26]. To this end, we trained the model on N–1 samples of the data and made a prediction on the one sample that was left out. This produces “out of sample” predictions for all N samples. These N predictions were pooled to compute various classification metrics, like the receiver operator characteristic (ROC), the area under the curve (AUC) of the ROC, and a confusion matrix of true versus predicted labels [27].

In addition, we used the Tree Shapley Additive Explanation (SHAP) algorithm [28], an explainable machine learning technique, on the random forest model to provide more interpretable predictions for each participant incorporated in the model. The Tree SHAP algorithm assigns each feature an importance value for every prediction. Each prediction begins at a base value, which is the expectation of the response variable (in our case, a probability between 0 and 1). Then, the SHAP values attribute to each feature the change in the expected model prediction when conditioning on that feature. The sum of the base value and all the additive feature attributions equal the final prediction probability.

All machine learning model development was performed using open-source packages in Python (Python Software Foundation, Wilmington, DE). The packages include but are not limited to Scikit-Learn, SHAP, Pandas, and Numpy.

#### Statistical Methods

Statistical analyses of changes in BP were performed using SAS software, version 9.4 (SAS Institute, Inc, Cary, NC). Change of continuous variables over time was analyzed using a two-tailed paired Student *t* test with alpha set at .05 and chi-square tests for differences in categorical variables. We used mixed-effects modeling to test the effects of baseline body mass index, baseline SBP, age, and gender on the mean change in BP.

## Results

### Intervention Participants

We identified 172 participants with hypertension (baseline BP≥130/80 mmHg or reported use of an antihypertensive medication) who engaged with the intervention for at least 2 weeks, reported a follow-up BP value, and were included in the *Primary* cohort. Demographics and baseline measurements for each cohort are presented in Table 1. There were no statistical differences in the baseline characteristics between those in the primary cohort and those in the two subgroups, as described above (ie, those who completed the intervention and those who completed and had a longer BP-tracking duration).

### Blood Pressure

In the *Primary* cohort, 75.0% (129/172) of participants had a clinically meaningful improvement in BP (defined as a decrease of ≥5 mmHg in SBP or ≥2.5 mmHg in DBP). The mean change from baseline to last follow-up reported was –11.5 mmHg for SBP and –5.9 mmHg for DBP, with a mean duration between values of approximately 9 weeks (62.6 days). An improvement of one BP category, as defined by ACC/AHA clinical practice guidelines [1], was seen in 51.7% (89/172) of the primary cohort. The changes between the end values of SBP and DBP were found to be significantly different from the corresponding baseline values (*P*<.001). The difference remained significant in the mixed-effects model accounting for the baseline SBP, age, gender, and body mass index (*P*<.001). The percent weight change was not found to correlate with changes in SBP (*P*=.53) or DBP (*P*=.12). Table 2 presents the changes in BP for the three analysis cohorts.

The mean duration between the baseline and final BP values for the *Completed Intervention* cohort was 10 weeks, with 74.7% (106/142) showing a meaningful improvement in BP and 22.5% (32/142) achieving a normal BP (BP<120/80 mmHg). The mean duration for the *Completed with Longer Tracking* cohort was 12.3 weeks, with 82.6% (71/86) of participants showing meaningful improvement and 26.7% (23/86) ending the intervention with BP in the normal range. The percentage of participants with meaningful improvements in BP was higher in this cohort than the *Primary* cohort (*P*=.02).

**Figure 3:**
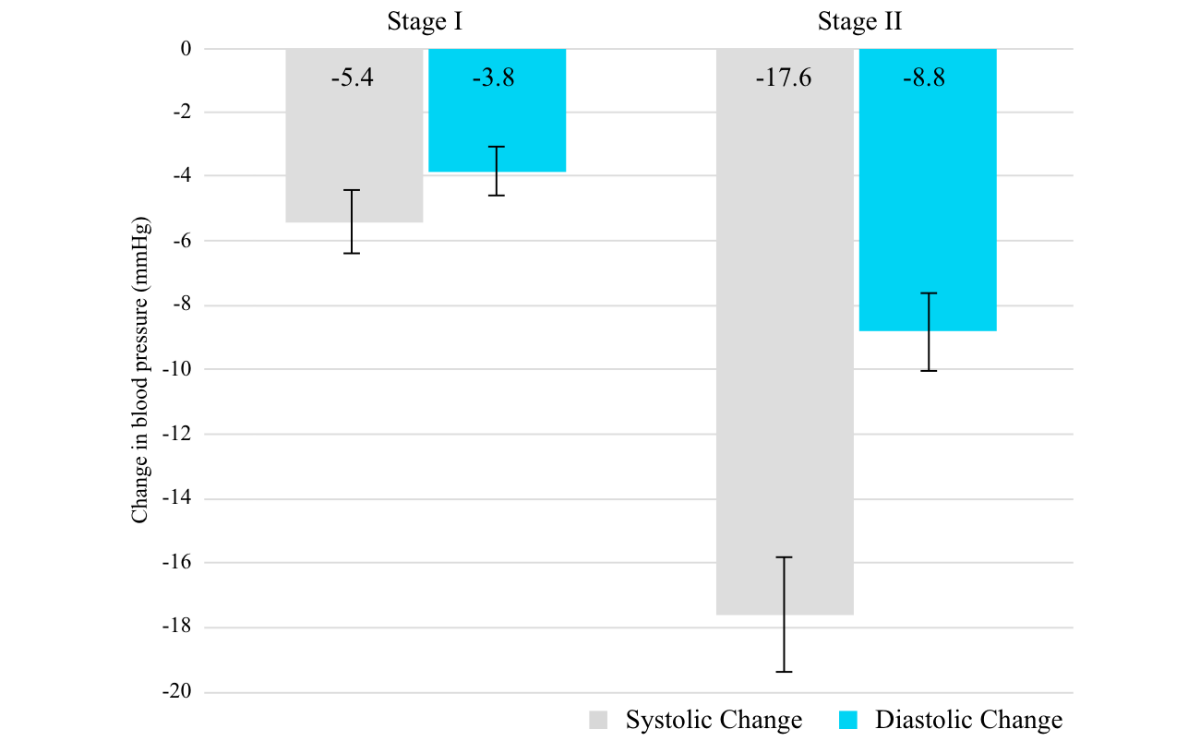
Observed change in participant blood pressure by baseline category.

Figure 3 contrasts the improvements seen in participants with stage I and stage II hypertension. Participants with stage I hypertension (n=76) saw a decrease of 5.4 mmHg (95% CI –7.4 to –3.3) in SBP and a decrease of 3.8 mmHg (95% CI –5.3 to –2.3) in DBP. Participants with stage II hypertension (n=84) observed a larger decrease in BP values, with SBP decreasing by 17.6 mmHg (95% CI –21.2 to –14.1) and DBP decreasing by 8.8 mmHg (95% CI –11.3 to –6.4).

**Figure 4:**
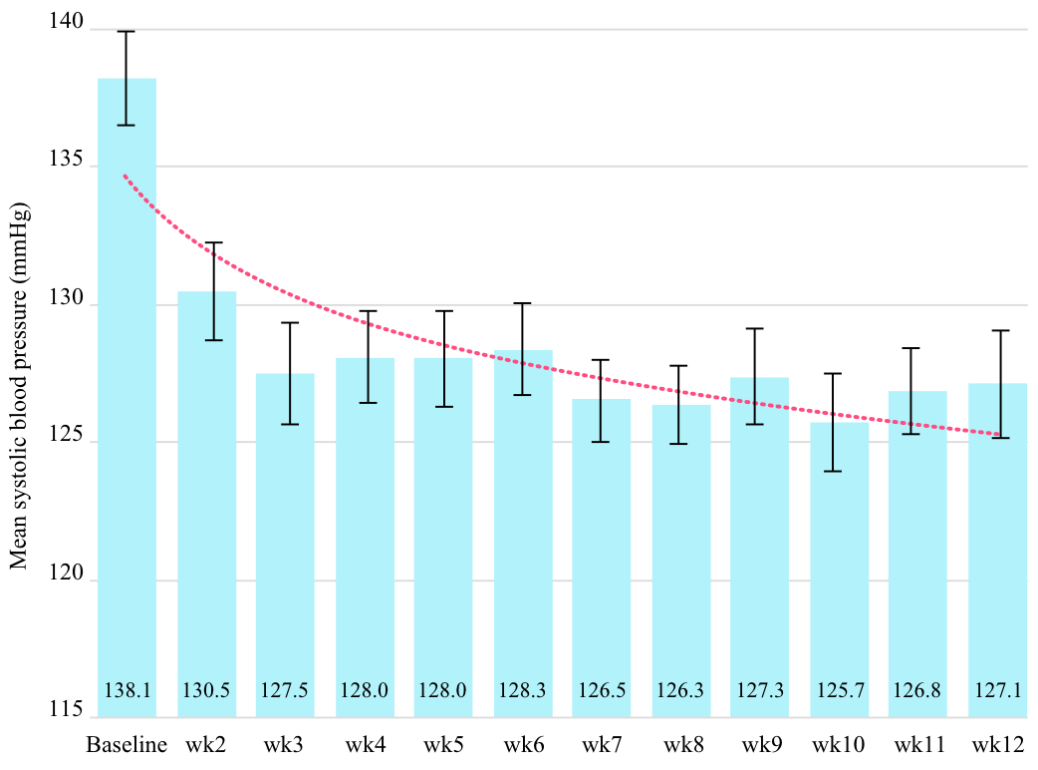
Mean systolic blood pressure over time in the Completed with Longer Tracking cohort. Plot of mean systolic blood pressure per intervention week, with SE bars. The sample size of weekly means varied from 42 to 86 participants.

Mean weekly SBP values from the *Completed with Longer Tracking* cohort were used to explore the rate of BP change (Figure 4). Although the mean BP continued to decline throughout the intervention period, the rate of decline was approximately 5 times greater in the first 6 weeks than in the following 6 weeks.

**Table 1:** Sample characteristics at baseline by intervention completion.

Participant characteristics	Primary cohort (N=172)	Completed intervention (N=142)	Completed with longer tracking (N=86)	*P* value^a^
Age^b^ (years), mean (95% CI)	55.0 (53.7-56.2)	55.0 (53.7-56.4)	55.1 (53.2-56.9)	.87
Body mass index (kg/m^2^), mean (95% CI)	35.3 (34.0-36.6)	34.9 (33.5-36.2)	34.3 (32.7-35.9)	.15
Female gender, n (%)	148 (86.1)	125 (88.0)	75 (87.2)	.66
Geographic distribution^c^ (number of US states)	28	28	23	.77
Systolic BP^d^ (mmHg), mean (95% CI)	138.9 (136.6-141.3)	138.6 (136.0-141.2)	138.1 (134.7-141.5)	.49
Diastolic BP (mmHg), mean (95% CI)	86.2 (84.8-87.7)	86.1 (84.5-87.7)	87.4 (85.3-89.4)	.12
Number of BP medications, mean (95% CI)	1.3 (1.2-1.5)	1.3 (1.1-1.5)	1.2 (0.96-1.5)	.12

^a^*P* value comparing the primary cohort to participants completing the intervention with longer tracking.

^b^Age was not available for 5 participants.

^c^US state data were not available for 50 participants.

^d^BP: blood pressure.

**Table 2:** Change in blood pressure across sample cohorts.

Measures	Primary cohort (N=172)	Completed intervention (N=142)	Completed and longer tracking (N=86)
Systolic BP^a^ change (mmHg), mean (95% CI)	–11.5 (–13.7 to –9.3)	–11.2 (–13.6 to –8.8)	–12.7 (–16.0 to –9.5)
Diastolic BP change (mmHg), mean (95% CI)	–5.9 (–7.3 to –4.4)	–5.8 (–7.5 to –4.1)	–7.4 (–9.7 to –5.1)
BP duration (days), mean (95% CI)	62.6 (58.4 to 66.8)	68.5 (64.1 to 72.8)	86.5 (84.2 to 88.7)
Number of average weekly BP readings^b^, mean (95% CI)	2.7 (2.4 to 3.1)	2.8 (2.4 to 3.2)	3.2 (2.6 to 3.7)
Meaningful changes in BP, n (%)	129 (75.0)	106 (74.7)	71 (82.6)
Follow-up BP average<140/90 mmHg, n (%)	132 (76.7)	108 (76.1)	69 (80.2)
Follow-up BP average<130/80 mmHg, n (%)	63 (36.6)	52 (36.6)	37 (43.0)
Follow-up BP average<120/80 mmHg, n (%)	39 (22.7)	32 (22.5)	23 (26.7)

^a^BP: blood pressure.

^b^Meaningful change is defined as a minimum decrease of 5 points in systolic blood pressure or 2.5 points in diastolic blood pressure.

### Engagement

In the *Primary* cohort, 94.2% (162/172) of the participants were using the app after 6 weeks (mid-intervention) and 82.6% (142/172) of the participants completed the intervention. Full app use data were available for 94.8% (163/172) of the participants in the *Primary* cohort. Use of the app was defined as active use of any feature, excluding the act of a login or logout. Total distinct app engagements averaged 12.2 per day (95% CI 10.9-13.4), and the average number of calls completed with an intervention program health coach was 3.4 (95% CI 3.1-3.7).

### Predictive Modeling

**Figure 5:**
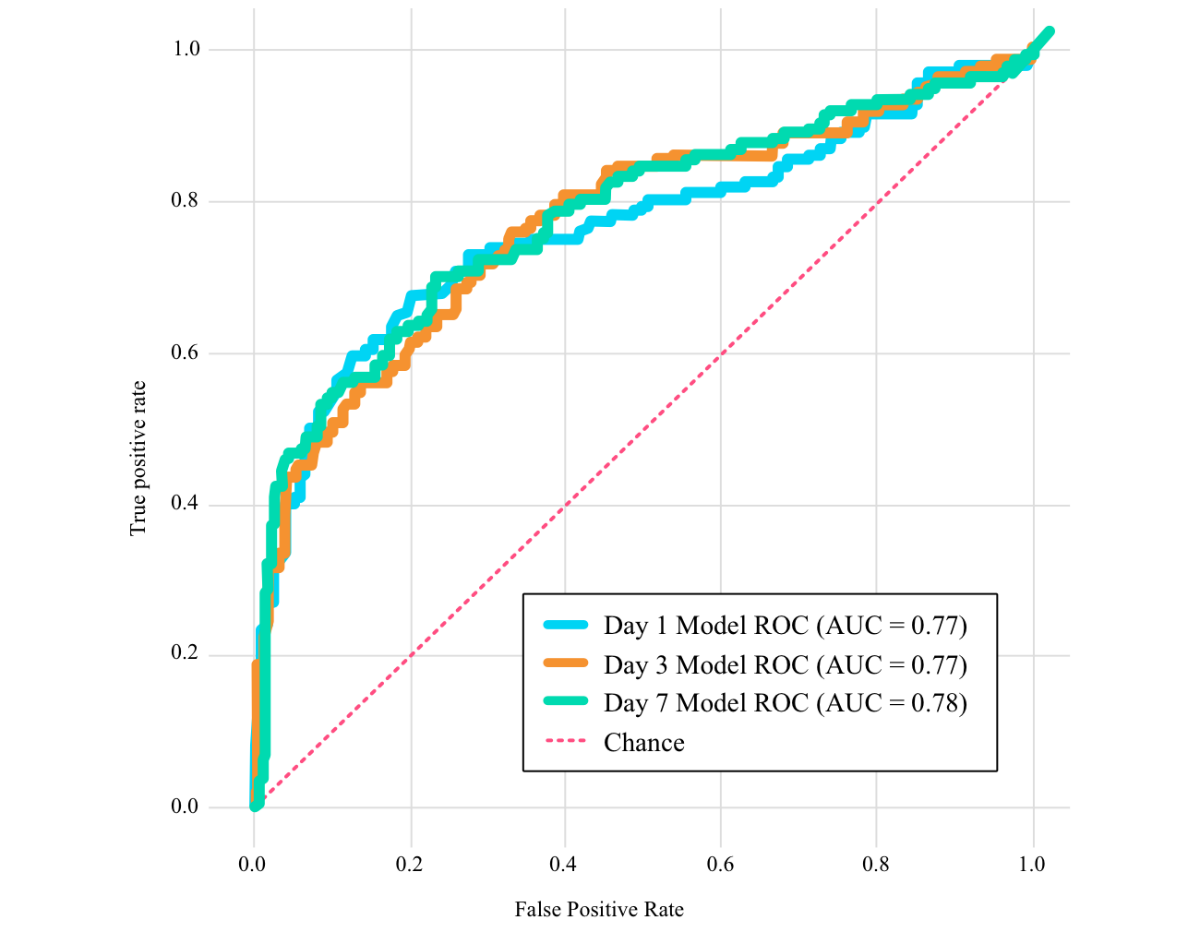
Receiver operating characteristic curves for predictive models of days 1, 3, and 7. ROC: receiver operating characteristic; AUC: area under the curve.

The random forest model was trained on 427 participants (*Prediction Model* cohort in Figure 1). The resultant ROC curve and AUC for days 1, 3, and 7 models showed that the model performs better as more days of data are used (Figure 5).

Performance of the day 7 model was examined with a target false positive rate of 25%. The nearest point on the ROC curve to the desired false positive rate was at 26%. At this point in the curve, we observed a sensitivity or true positive rate of 70% and a specificity or true negative rate of 74%. The observed misclassification rate or error rate was 27%. The positive predictive value was 56%, and the negative predictive value was 84%.

The Tree SHAP algorithm was applied to ascertain which model features best predicted intervention completion in the entire analyzed population (Figure 2). The results indicate that, on an average, early engagements directly related to intended behavioral interactions and changes (eg, self-monitoring of biometrics, completing supportive and core app interactions, or reporting more exercise) are most predictive of intervention completion; sociomarkers (eg, Android vs iPhone use) are also predictive but to a lesser degree.

**Figure 6:**
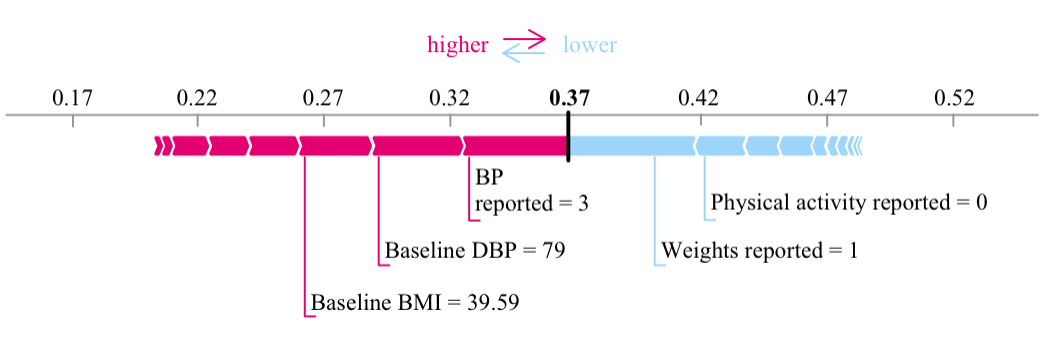
The day 7 probability of intervention completion for one participant, represented by the Shapley Additive Explanation Force Plot. Feature use contributing to a higher probability of intervention completion is shown in red, along with the size of the feature’s contribution. Feature use contributing to a lower probability of intervention completion is shown in blue. DBP: diastolic blood pressure; BP: blood pressure; BMI: body mass index.

To illustrate how the machine learning model can convey both a completion probability and the contribution of each feature to that computed probability for a single participant, a plot of the SHAP values for a random participant at day 7 is shown in Figure 6. In this example, the participant’s tracking of BP and baseline body mass index contributes to a higher probability of completion, but this is partially counteracted by the lack of reporting in exercise and relatively low tracking of weight.

## Discussion

### Principal Findings

The digitally delivered intervention resulted in meaningful reductions in BP in adults. The majority of BP reduction was observed within the first 6 weeks of the intervention, indicating a rapid response to the digital intervention. By the end of the 12-week intervention, a high proportion of participants achieved BP control as per the ACC/AHA definition (43% in the *Completed with Longer Tracking* cohort) [1]. The greatest reductions were found in participants with stage 2 hypertension, with a mean SBP improvement of 17.6 mmHg. The BP-lowering effects observed were comparable or greater than those observed in other digitally delivered multicomponent interventions [14-16,24,29].

We did not find evidence that these improvements were the result of intensified medication therapy or that the BP reduction was due to weight change alone. This suggests that behavioral changes made during the intervention period account for much of the reduction in BP observed, and this observation is consistent with the effect sizes of other intensive behavioral or lifestyle interventions [29-32]. Importantly, the effects observed here are meaningfully greater than those observed by self-monitoring of BP alone, which suggests that multiple behaviors contribute to the effects [29,33]. For example, the 2017 meta-analysis conducted by Tucker et al showed that self-monitoring of BP was associated with changes of –3.2 mmHg in SBP and –1.6 mmHg in DBP between baseline and 12-month clinic measurements as compared to usual care [33]. In other analyses (data not shown), we did not find any correlation between the degree of self-tracking and BP change, nor did we find any difference between participants who were provided a home BP cuff and those who already had one.

A proof-of-concept analysis of a predictive model developed using machine learning demonstrated the ability to predict intervention completion after just one full day of engagement. The ability to predict intervention completion in a timely fashion is important for several reasons: (1) Given the typical patterns of apps use, there is likely a short time period during which an intervention adjustment can be made to increase completion rates. (2) Ongoing participation in the intervention is associated with a very high probability of achieving meaningful BP reductions. (3) Completion may be important for sustainment of behavioral changes and resulting outcomes beyond the intervention period.

This type of machine learning model can be implemented by choosing an operating point at which to make predictions. The operating point is chosen based on the balance of false versus true positives that it is expected to create. The prediction of likely to complete or not to complete the intervention can then be acted upon by leveraging SHAP values and creating an automated set of actions such as providing tailored feedback, reinforcement, warnings, and reprioritization of behavioral goals. The value of the model can then be studied in this context to see how it alters both completion rates and clinical outcomes.

This prediction methodology creates the opportunity for other exciting applications that may further improve the effectiveness of treatment. For example, the same methodology can be used to predict more direct measures of treatment success, such as a specific degree of BP improvement. Once a model that predicts clinical outcomes in the midst of treatment is validated, it can be used to alter the course of treatment with the intent of improving outcomes and patient experience.

Finally, machine learning allows us to explore discrete components of a digital intervention and the way they interact with participant characteristics. For example, in the current model, we found that the count of exercise sessions reported in the first week of the intervention was highly predictive of intervention completion. We also found that novel sociomarkers such as email domain or phone operating system had predictive capacity. For example, participants who used Yahoo email were more likely to complete the intervention than users of other email domains. It may be that Yahoo email use is a proxy for older age and other personality or socioeconomic features [34].

### Limitations

A meaningful limitation of this retrospective analysis is the lack of a control group to evaluate the true effect size of this behavioral intervention. However, the effect size observed can be compared to similar study cohorts reported in the literature. For example, in a recent 8-week study comparing metformin to placebo in nondiabetic adults with hypertension having similar demographic features and baseline BPs, the control group (n=49) had a mean improvement of 2.6 mmHg in SBP when measured in the clinic and a 0.7 mmHg mean increase when measured with 24-hour ambulatory BP monitoring [31]. Larger improvements of 6.0 mmHg in SBP were seen in the control group (n=131) in a 12-month study comparing the impact of electronic counseling on the standard of care for BP in adults with hypertension [29]. Participants of that study were recruited from the Heart and Stroke Canada website, and the authors hypothesized that this may have resulted in a study cohort of independently motivated participants, where participants assigned to the control group were more likely to take action with the standard of care recommendations. Improvements in that control group were clinically meaningful but are about half the size of those observed in our study cohort.

A limitation of our machine learning model is the size of the training dataset used, which typically correlates with the predictive strength of the model and limits the number of features that can be explored. However, it is encouraging to see that predictive power and feature importance can emerge from a relatively small dataset. This should encourage others to begin using machine learning models early, rather than waiting for massive datasets to accrue. The strength of the any machine learning model can be expected to improve over time as the training dataset grows.

### Conclusions

Reductions in BP were observed among adults with hypertension who use the digital therapeutic studied here. The degree of BP reduction was clinically meaningful and achieved rapidly by a majority of participants studied. Greater improvement was observed in participants with more severe hypertension at baseline. A successful proof of concept for using machine learning to predict intervention completion after one day of app use was presented. Future research should examine the ability of treatment tailored in response to this model to further enhance outcomes. In addition, research is needed to assess the durability of outcomes following the intervention period, to identify subgroups and subgroup characteristics where the targeted intervention is most/least effective, and on the use of machine learning to predict clinical outcomes and modify treatment parameters during the course of treatment. The digital intervention should also be evaluated for its effectiveness in treating other chronic diseases that share the same root causes as hypertension.

## Abbreviations

ACC: American College of Cardiology
AHA: American Heart Association
AUC: area under the curve
BP: blood pressure
DBP: diastolic blood pressure
ROC: receiver operator characteristic
SBP: systolic blood pressure
SHAP: Shapley Additive Explanation
